# Social media use in higher education: Building a structural equation model for student satisfaction and performance

**DOI:** 10.3389/fpubh.2022.1003007

**Published:** 2022-09-20

**Authors:** Ali Mugahed Al-Rahmi, Alina Shamsuddin, Eta Wahab, Waleed Mugahed Al-Rahmi, Ibrahim Yaussef Alyoussef, Joseph Crawford

**Affiliations:** ^1^Faculty of Technology Management and Business, Universiti Tun Hussein Onn Malaysia, Parit Raja, Malaysia; ^2^Faculty of Social Sciences & Humanities, School of Education, Universiti Teknologi Malaysia, Skudai, Malaysia; ^3^Faculty of Education, Education Technology Department, King Faisal University, Al Hofuf, Saudi Arabia; ^4^Faculty of Education, University of Tasmania, Launceston, TAS, Australia

**Keywords:** user acceptance, task-technology fit, social media, technology acceptance model, performance expectancy, performance impact, higher education, behavioral intention

## Abstract

Social media utilization at the student-level has become more prevalent contemporary higher education. Hence, this study is aimed at developing a specific model, along with the behavioral intention to use, to explore educational quality, actual social media use, and task-technology fit that affects student satisfaction and performance impact through examining the synergies of constructivism, user acceptance and usage of information technology, and technology acceptance. To test, a survey was administered to 430 students across five Malaysian universities. Through structural equation modeling, findings indicate that to improve student satisfaction and student performance through embedded social media, students need to have opportunities to collaborate on learning, have easy access to social media, perceive such use to be easy, and have aligned expectation on performance and effort. Interestingly, the actual social media use, was the only variable in the model that did not predict student satisfaction, despite its role in predicting student performance. The study highlights that constructivist learning, as well as task-technology fit over social media, enhances the students' learning experience and enables knowledge sharing and dissemination. The effect of using social media on student satisfaction and academic performance highlights that all students think that it is adequate for their instructors to improve their usage of social media tools. Therefore, we advocate learners and students employing social media for academic purposes with the help of lecturers at higher teaching organizations and institutions.

## Introduction

The COVID-19 pandemic has led to rapid adoption of digital technology, particularly during government-enforced lockdowns and social distancing regimes. This is particularly relevant for higher education, with universities required to respond to enable continuity of learning despite rapid digitalization activities (1, 2). Embedded educational technologies have now likely become more normal than an alternative in a post-pandemic world, with educators seeking to innovate their Ed Tech practices to better enable their emergency remote teaching and beyond (3). Accordingly, this paper focuses on the postgraduate student use of social media. Social media while having inherent benefits accessible, highly interactive, networking, stimulation (4) it offers a unique opportunity to strengthen higher education learning and teaching by creating more accessible and temporarily situated learning content. Existing evidence supports the role of social media in tertiary student educational performance (5, 6). Increasingly, as social media adoption in class has increased, so too has the pedagogical literature (7, 8).

Social media use in higher education can enable greater comprehension of others' intentions and support behavioral management, specifically for collaborative learning as students can interact in both synchronous (e.g., FaceTime or live commenting/messaging) and asynchronous means (e.g., delayed responses to group posts) (9, 10). Social media is gaining momentum as the total users are increasing, with 2.8+ billion Facebook users, and 2.2+ billion on YouTube. The bricolage effect also leads individuals to generate collective meaning through interactions across multiple social media channels. With this in mind, learners are utilizing their perceived appropriate media type for a task. For instance, TikTok for short video-based work and Facebook or WeChat for managing group conversations and meetings (11, 12). The opportunity for knowledge-sharing notwithstanding, students have the capacity to use such resources for knowledge generation, external feedback, and learning opportunities. Importantly, the use of social media by students (current and prospective) highlights the opportunity to better engage with technologies that students are familiar with by integrating and embedding it into curriculum (13). The impact on student educational attainment may initially be dependent on their usage and adoption of social media (14, 15). However, numerous studies highlight that the use of social media, aside from academic success and performance among students at the tertiary level, is positively (8, 16–18). The challenge and complexity remains in how social media usage, fit, and adoption affect how students perceive their experience (e.g., student satisfaction) and how they perform (e.g., academic achievement). This manuscript seeks to better understand the underlying constructs that predict both of these outcomes.

To elaborate, this research aims to examine how student satisfaction and academic performance were predicted by technology (e.g., behavioral intention to use, actual social media use, and task-technology fit) and pedagogy (e.g., constructivist learning). Notwithstanding the existing literature that relates social media to academic performance and satisfaction, the context and construction of this study is novel. First, while dominant literature emphasizes developed Western nations (e.g., United Kingdom, United States, and Australia), this study is situated in Malaysia where social media embedding remains in its infancy. Second, while many studies draw on technology-, behavior- or pedagogy-based predictors, this study emphasizes both and their interaction. Importantly, this study recognizes that humans operate technology bringing their previous experience and expectations to the way they engage with such technology. In lesser developed nations, the use of social media is less pronounced. This study seeks to integrate pedagogical, behavioral and technological predictors to better understand how learning and teaching using social media can be enhanced.

This study investigates ways social media can be employed to create a better educational experience for students through collaborative learning and task-technology fit. Using social media for behavioral purposes, with perceived usefulness and usability, and its consequences in connection with the students' actual social media purpose is also based on students' perceptions of their performance expectations and their efforts to use social media, which sequentially increase student satisfaction and impact on academic performance. The technology acceptance model (TAM) is one of the most widely adopted behavioral models for the usage of social media. The theoretical model proposed in this work draws on TAM, alongside the unified theory of acceptance and usage of technology (UTAUT), task-technology fit, and constructivist learning. The model proposed theorizes that the interaction of TAM, UTAUT, and constructivist learning will predict student satisfaction and academic performance. This research was designed to provide a model for finding critical aspects that would play a significant role in the behavior of students using social media for quality of education, actual social media use, and task-technology fit to increase their performance in education at higher education.

This paper presents three new key insights on the influence of social media on students' intention to use it through educational quality, actual social media use, and task-technology fit to increase students' satisfaction and academic performance impact by: (i) investigating the factors influencing learners' behavioral intention toward using social media through educational quality, actual social media use, and task-technology fit; (ii) finding the interactions among all elements; and (iii) building a new model on the students' behavioral intention to use social media through educational quality, actual social media use, and task-technology fit to improve their performance impact in higher education. In essence, our research goal is to research and assess the behavioral intention of students to use social media through educational quality, actual social media use, and task-technology fit in order to improve their educational outcomes in higher education.

### Research question

What technology-use, student behavior, and pedagogy factors collectively affect student satisfaction and academic outcomes?

### Problem statement

Information and communications technologies such as social media tools in mainstream education have transformed traditional teaching and learning in higher education (19, 20). The use of social media has an impact on academic performance, grade point averages (GPA), and educational achievement (5, 20, 21). With such inclusion has been growing work evaluating the efficacy of embedding social media technologies on educational outcomes. Many of these focus on technological, pedagogical, or individual differences characteristics in isolation. This manuscript responds by theorizing and empirically testing a model which encompasses technology acceptance, constructivist learning, user acceptance and use of technology, and task-technology fit theories as collective predictors of student success (e.g., academic performance and student satisfaction). This is increasingly important, with emergent evidence proposing that social media use may negatively affect educational performance (22–25). Malaysia, as a developing Eastern nation, offers a unique and positive context to situate this model testing in. According to previous models, both perceptual and interaction constructs have been empirically evaluated, without considering task-technology fit (26, 27). Hence, the objective of this research is to understand the synergies of a holistic model of technology acceptance (28, 29), constructivist learning (30, 31), use of technology (28), and task-technology fit (32) on higher education student outcomes (e.g., success and satisfaction) in Malaysia.

## Research model and hypotheses development

**Figure 1:**
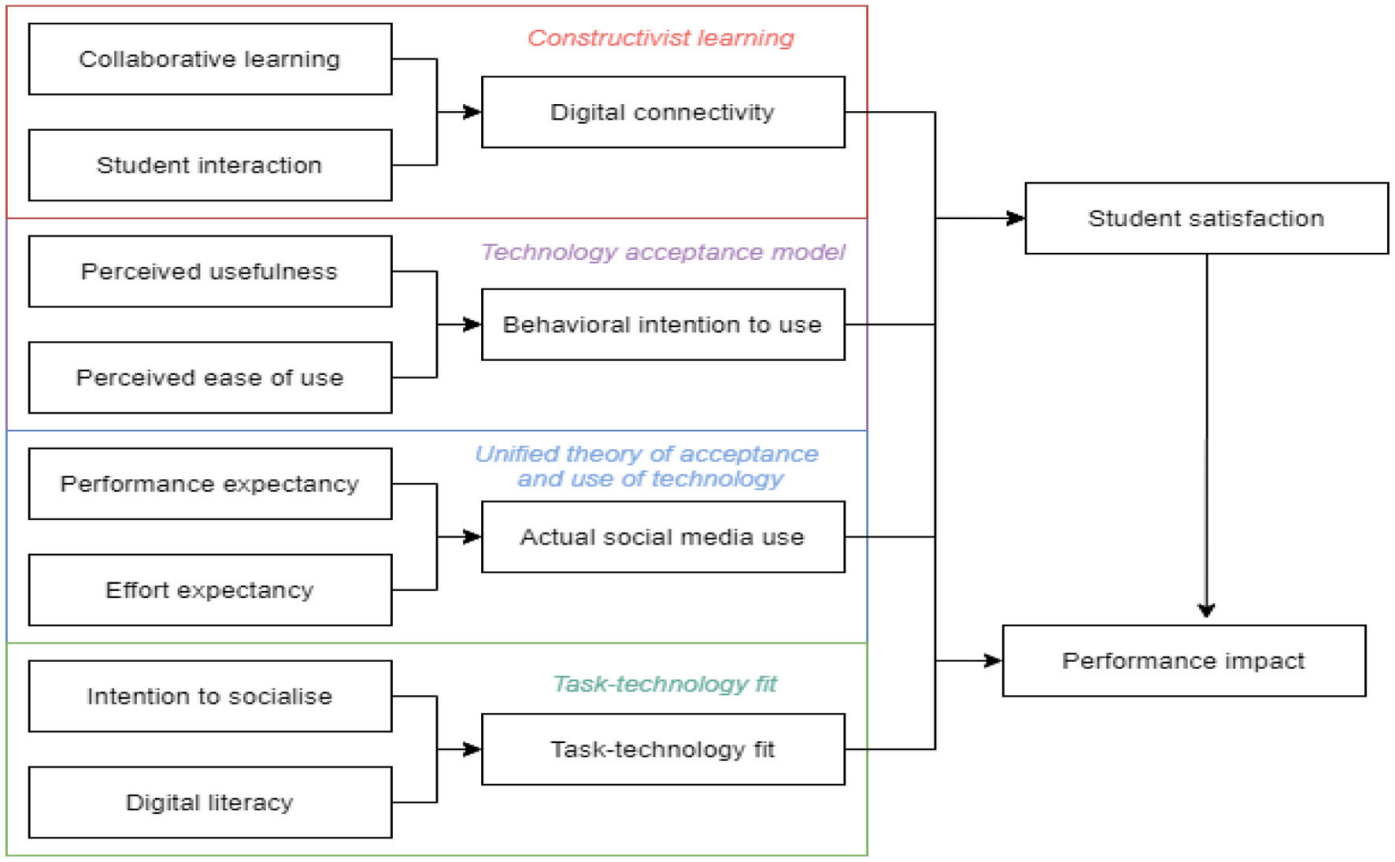
Research model and hypotheses.

The research model (see Figure 1) comprises constructivist learning in a digital context (e.g., collaborative learning, student interaction, and digital connectivity), technology acceptance (e.g., perceived usefulness, perceived ease of use, and behavioral intention to use), the unified theory of acceptance and use of technology (e.g., performance expectancy, effort expectancy, and actual social media use), and task-technology fit (e.g., task technology-fit, intention to socialize, and digital literacy). Importantly, this model brings together behaviors and perceptions of students (e.g., intention to socialize with other students online), the skills and knowledge they have with digital technology (e.g., digital literacy), and their technology use, and how each of these effect student satisfaction and performance. Further developing this point, it also proposes a generalizable comprehensive model grounded in the effect that social media and student interaction through social media (33) has on students' educational performance. To further explain the proposed model, each relationship is grounded in the literature following with appropriate hypotheses established.

### Collaborative learning

Collaboration is when a group of individuals work together as a group connected or consistent to enhance the attainment of a specific purpose or end product (34). Similarly, collaboration is considered as a personal lifestyle and interaction philosophy where individuals are accountable for their activities, such as learning and appreciating the skills and contributions of their peers (35). Collaboration in learning and teaching is an educational strategy involving learners collaborating to resolve specific problems or complete a task (36, 37). Social media works positively and increases the academic achievement of students (38, 39). Furthermore, the amount of perceptual work imposed on the intellectual capabilities of students must be the key factor ensuring whether they prefer collaboration or working individually to be used for learning purposes (40, 41). This research suggests that the constructivism theory of collaborative learning influences instructors' use and willingness to use social media in teaching and learning. Thus:

***Hypothesis 1***. Collaborative learning will have a positive effect on digital connectivity (on social media).

### Students' interaction

Web-based instruction is a dynamic online media platform that enables users in collaboration and remote contexts to communicate synchronously and asynchronously (42). Traditional learning techniques in research group members may affect friendly conversations (43). As a result, communication has an impact on learning skills (30). Facebook, as one example, is an efficient platform for increasing learners engagement in eLearning information, enabling active learning with a greater eagerness to gain information, and have higher quality knowledge sharing opportunities with other students, and generate a sense of belonging (4, 44). This study proposes that students who interact together will also have a desire to do so in digital contexts. Thus:

***Hypothesis*** **2**. Student interaction will have a positive effect on digital connectivity (on social media).

### Perceived usefulness

Perceived usefulness refers to the extent a person believes it is possible to improve their efficiency by employing specific systems (28), and is defined in this study as the extent to which a learner believes that using social media would improve their learning performance (45). Perceived usefulness refers to the likelihood that users subjectively perceive that the use of such a system application in an organization increases their performance at work (28, 46). As David and Hansen et al. (28, 45) discovered, perceived usefulness was found to be an essential determinant of intention-to-use behaviors. Previous research relates perceived usefulness to usage behavior (47), and this hypothesis is maintained in this research in the context of social media.

***Hypothesis 3***. Perceived usefulness will have a positive effect on behavioral intention to use social media.

### Perceived ease of use

Perceived ease of use refers to when individuals believe using a given method will require less effort (28) and in context, can be defined as the degree to which a student perceives social media use as easy and would improve their learning performance. In the information technology literature, empirical research has been utilized to examine and validate the link between attitude components and perceived ease of use (38). Existing research generates consistent arguments, suggesting that the two technology acceptance model concepts are substantially associated with attitude (48–50).

***Hypothesis 4***. Perceived ease of use will have a positive effect on behavioral intention to use in using social media.

### Performance expectancy

Performance expectancy is “the degree to which a person believes it is possible to improve their efficiency employing specific systems” (28, 29). The unified theory of acceptance and usage of technology (28, 29) posits performance expectancy as an important foundation concept of behavioral intention. The literature demonstrates that performance expectancy is a predictor of behavioral intention (29, 51, 52). In this study context, this translates to social media use, in that when students expect performance gain from using social media, they will in turn have higher actual use. The current research postulates that there is a relationship between performance expectancy and the behavioral intention to use them.

***Hypothesis 5***. Expected performance will have a positive effect on actual social media use.

### Effort expectancy

Embedded social media in higher education is not new (53), with the benefits becoming more self-evident. The unified theory of acceptance and usage of technology suggests that effort expectancy is one of the direct elements of behavioral intention. Several articles describe how effort expectancy is a major factor in behavioral intention. For example, Howard et al. (54) examined the peer intentions of pre-service teachers toward using unified theory of acceptance and usage of technology in information technology and discovered effort expectancy as a major predictor of behavioral intention toward information technology (55). The current research will show that there is a relationship between effort expectancy and behavioral intention to use.

***Hypothesis 6***. Effort expectancy will have a positive effect on actual social media use.

### Intention to socialize

Intention to socialize is used as mediators for the study of the relationship between the inputs and outcome variables for past social media research. According to Perrow (56) used social effects, user contributions and their intentions to socialize are different across virtual communities. Other studies have employed social capital as a mediator to demonstrate user objectives and behavior. For instance, the importance of trust to developing individuals' readiness to accept something or somebody was studied by Chung et al. (57), while Dholakia et al. (58) and Chen and Tseng (59) and Hsiao (60) reviewed the social interactions and evasive effects of cohesiveness mediation in the team. As a mediator, Ahmed et al. (61) explored user participation in virtual companies' social impact. Therefore, apart from positive attitude, another construct that is anticipated to have a causal relationship that increases social media use, task-technology fit, is social characteristics. Based on the above discussion, the following hypothesis was proposed:

***Hypothesis 7***. Intention to socialize will have a positive effect on task-technology fit.

### Digital literacy

Digital literacy refers to the level at which a technology has the features to achieve what is regarded as suitable (62). A range of accessible social media technologies such as Twitter, Facebook, and LinkedIn are intended and constructed for distinct user groups' individual objectives and aims. The selection of such instruments may demonstrate the effects on expected behavior of causal priming (63). We examined the extent to which students understood elements of social media. Task functionality was compared to task performance, and the use of media was found to benefit task performance (64). The degree to which the incorporation of information and system quality tooling affects anticipated results was studied by Cheng and Tseng (59). According to Koo et al. (65) employed the task-technology fit model to investigate the elements that affect personal performance in business resource planning. Moreover, Wang and Lin (66) revealed task-technology fit impacts and team performance distribution in repeated jobs. Two kinds of technical elements were utilized in their frames. Therefore, apart from positive attitude, another construct that is anticipated to have a relationship that increases social media use, task-technology fit, is digital literacy.

***Hypothesis 8***. Digital literacy will have a positive effect on task-technology fit.

### Digital connectivity

Digital connectivity is defined as the degree to which the higher educational system has been successful in supporting student learning arrangements through collective learning in digital contexts (67). Quality online education ideals can be found to match well with the fundamental concepts of effective student education (68). The content value on social media and the internet is solely dependent on the student use of social media tools for teaching and learning. Though there may be actual peer-review to address false and/or erroneous material, the efficacy may vary. Sutherland and Jalali (69) has found a need for stronger evidence and evaluation of student results when considering connectivity in education. Some research required social media tools to establish a moderating service to evaluate the quality of educational videos, but one study commented that, considering the volume of new content published on YouTube every day, this was probably not a viable idea (70). Digital connectivity, therefore, is assumed to have a positive effect on behavioral intention to use, students' satisfaction, and performance impact. It is assumed that such environmental conditions will have a positive effect on the intention to use as well.

***Hypothesis 9***. Digital connectivity will have a positive effect on behavioral intentions to use social media.
***Hypothesis 10***. Digital connectivity will have a positive effect on student satisfaction in using social media.
***Hypothesis 11***. Digital connectivity will have a positive effect on performance when using social media.

### Behavioral intention to use

Behavioral intention to use is defined as the willingness of the person to continue or utilize technology, including elements that affect technology use intentions (29). For this study, behavioral intention is the degree to which learners will use social media in the future to study together. Thus, this study investigates students' behavioral intentions for consuming social media for virtual communications skills to enhance their learning performance. Additionally, this research identifies social media use for greater association and learning as a critical factor in emerging technology-based theories (28, 71). All of these theories developed out of the core of the Theory of Reasoned Action TRA, which asserts that usage of social media is a function of one's attitude toward certain standards; later, the term “The theory of planned behavior TPB” was expanded to include seeming control (71, 72). In addition, as regular users believe, perceived usefulness and ease of use might be taken for granted, resulting in increased user satisfaction and continued purpose (73, 74). Thus,

***Hypothesis 12***. The behavioral intention to use will have a positive effect on actual social media use.
***Hypothesis 13***. Behavioral intention to use will have a positive effect on students' satisfaction in using social media.
***Hypothesis 14***. Behavioral intention to use will have a positive effect on performance impact when using social media.

### Actual social media use

There is a strong relationship between the behavioral intention to use technology for learning and the actual usage of it (20). Yet, behavioral intention to use social media is rather low on the actual usage of social media to learn by students. Perhaps, this is due to social media being less widely accepted for academic use (75, 76). It, however, can be a valuable educational technology by enhancing social interactions, connectivity between peers, and support diverse relationship bonds forming (77, 78). Thus, social networks have both positive and negative effects on students, and the ultimate impact on a student is determined by their behavior (79, 80). The current research posits that there is a relationship between actual usage and student satisfaction and performance impact.

***Hypothesis 15***. Actual social media use will have a positive effect on student satisfaction.
***Hypothesis 16***. Actual social media use will have a positive effect on performance impact.

### Task-technology fit

Task-technology fit is defined as the degree to which systems match interests, suit or fit tasks, and meet requirements (81–83). In terms of the use of technology in organizations, actual use is inadequate for presenting a complete report without taking task technology into full deliberation; that is, if the technologies fit their conforming tasks (81, 84). A number of articles have investigated the beneficial impacts of task technology fit on usage behaviors, including on socializing with others and performance (84–86). In this study, the effects of task-technology fit are examined grounded in the direct and validated effects of actual use and users' satisfaction on measured education sustainability and on measured sustainability of training (52, 87), as well as the impact of actual use and user content on performance.

***Hypothesis 17***. Task-technology fit will have a positive effect on actual social media use in higher education.
***Hypothesis 18***. Task-technology fit will have a positive effect on student satisfaction.
***Hypothesis 19***. Task-technology fit will have a positive effect on performance impact.

### Student satisfaction

The students are happy and satisfied with their experiences (88, 89). Two characteristics are deemed crucial and important in the application of specific technologies by the user and their satisfaction: the perceived usefulness and ease of use. These characteristics are significant as user satisfaction with a technology is predictable (28, 90). User experience is acknowledged as a predictor of performance. The satisfaction enabled through technology effects the intention to socialize, and future performance and user adoption (91). In this context, Dumpit and Fernandez (92) proposed to improve learning satisfaction of students through embedded online engagement. As a result, it is proposed that the satisfaction of a student will also affect their performance. The performance impact is the result of formal education where students, learners, facilitators, and institutes have accomplished their academic objectives (28). Regarding Shayan and Iscioglu (93), social media in the fields of scientific studies keeps manipulating learners' academic accomplishments. The outcomes of satisfied use of educational technology (including Twitter and Facebook) Sayaf et al. (94) supports student understanding and learning (88). Further developing this point (36), they endeavored to discover the connection between student educational performance effects and Facebook. Moreover, social media usage helps to construct a positive relationship between the users' academic performance and their satisfaction (94).

***Hypothesis 20***. Student satisfaction will have a positive effect on performance using social media.

## Research method

### Sample

This study comprised an online and physical survey distributed to 445 postgraduate students at five Malaysian universities. This was across diverse ethnicities and cultures creating the conditions for a generalizable study. 430 were sent back by participation (96.2% return rate), with 15 questionnaires excluded for incompletion. In the final sample, 296 participants (68.8%) were male, 134 participants (31.2%) were female. Most participants were younger (29 and below), with frequent engagement with social media, and from a science, technology, engineering, and mathematics (STEM) discipline. Table 1 provides specific breakdowns.

**Table 1:** Demographic profile.

**Sample characteristics**		***n***	**%**
Gender	Male	296	68.8
	Female	134	31.2
Age	18–20	31	7.2
	21–24	70	16.3
	25–29	147	34.2
	30–34	88	20.5
	35–40	60	14.0
	41–45	22	5.1
	46+	12	2.8
Discipline	Social science	68	15.9
	STEM	246	57.3
	Business	96	22.3
	Other	20	4.6
Social media use	Constantly logged on	183	42.6
	Several times per day	212	49.3
	Once per day	26	6.0
	Once in a few days	26	6.0
	More than twice a week	8	1.9
	Less than once per week	1	0.2

### Data collection

Furthermore, the second part of the study's information and data was analyzed using IBM's SPSS and the AMOS. The study considered the construct validity of the measures including convergent and discriminated validity. Moreover, it explored the structural model. This technique was preferred by Hair et al. (95). The survey comprised two sections: the first consisted of survey items, in which the demographic information were collected (gender, age, institution, and discipline) and the second section consisted of survey items for measures of constructivist learning, technology acceptance, the unified theory of acceptance and use of technology, and task-technology fit.

## Results and measures

### Part 1: Constructivist learning

Constructivist learning comprised collaborative learning, student interaction, and digital connectivity. To measure collaborative learning, two existing measures were adapted to create a composite tool (96, 97). The internal consistency was good (α = 0.78), with items demonstrating strong model fit (χ2 = 321.215; CFI = 0.916; TLI = 0.907; SRMR =0.089) and average variance explained below 0.80 to demonstrate discriminant validity. Student interaction was measured through a composite scale based on the literature (96, 97). The internal consistency was good (α = 0.88), with items demonstrating strong model fit (χ2 = 103.130; CFI = 0.914; TLI = 0.902; SRMR = 0.066) and average variance explained below 0.80 to demonstrate discriminant validity. Digital connectivity was measured through items adapted based on Bozanta and Mardikyan (98) and Wu and Chen (99). The internal consistency was good (α = 0.878), with items demonstrating strong model fit (χ2 = 175.920; CFI = 0.926; TLI = 0.913; SRMR = 0.074) and average variance explained below 0.80 to demonstrate discriminant validity. The confirmatory factor analysis (CFA) showed positive inter-dimensional relationships (between 0.43 and 0.58), and all items loaded to their latent construct between 0.72-0.88. The overall model showed good fit (χ2 = 608.074; CFI = 0.958; TLI = 0.932; SRMR = 0.080).

### Part 2: Technology acceptance model

The technology acceptance model was measured by perceived usefulness, perceived ease of use, and behavioral intention to use. Perceived usefulness was measured with existing items (98) and showed good internal consistency (α = 0.86), strong model fit (χ2 = 241.203; CFI = 0.917; TLI = 0.905; SRMR = 0.081) and average variance explained below 0.80 to demonstrate discriminant validity. Perceived ease of use was measured with composite items from Bozanta and Mardikyan (98) and Wu and Chen (99). These items highlighted robust internal consistency (α = 0.88), and strong model fit (χ2 = 137.350; CFI = 0.913; TLI = 0.900; SRMR = 0.0.072) and average variance explained below 0.80. The behavioral intention to use items were adapted from Bozanta and Mardikyan (98) and Wu and Chen (99), and showed good reliability (α = 0.72) and validity (χ2 = 246.364; CFI = 0.918; TLI = 0.906; SRMR = 0.076; AVE = 0.51). The technology acceptance model CFA demonstrated good suitability with interdimensional relationships positive (0.60–0.71), all items loaded to their latent construct between 0.47 and 0.88. The overall model showed good fit (χ2 = 761.527; CFI = 0.912; TLI = 0.898; SRMR = 0.089).

### Part 3: Unified theory of acceptance and use of technology

The unified theory of acceptance and use of technology was measured by performance expectancy, effort expectancy, and actual social media use. Performance expectancy was measured with an adaptation of items from Dang et al. (100) and Escobar-Rodríguez et al. (101) and showed good internal consistency (α = 0.86), strong model fit (χ2 = 19.723; CFI = 0.986; TLI = 0.973; SRMR = 0.029) and average variance explained below 0.80 to demonstrate discriminant validity. Effort expectancy was measured with composite items from Dang et al. (100) and Escobar-Rodríguez et al. (101). These items highlighted robust internal consistency (α = 0.90), and strong model fit (χ2 = 54.291; CFI = 0.961; TLI = 0.922; SRMR = 0.035) and average variance explained below 0.80. The actual social media use items were adapted from Larsen et al. (102), and showed good reliability (α = 0.79) and validity (χ2 = 106.846; CFI = 0.934; TLI = 0.917; SRMR = 0.055; AVE = 0.57). The unified theory of acceptance and use of technology CFA demonstrated good suitability with interdimensional relationships suitable (0.42–0.45), all items loaded to their latent construct between 0.72 and 0.87. The overall model showed good fit (χ2 = 721.851; CFI = 0.940; TLI = 0.910; SRMR = 0.42).

### Part 4: Task-technology fit

Task-technology fit was measured with quantitative questions on perceived task-technology fit, intention to socialize, and digital literacy. To measure perceived task-technology fit, two existing measures were adapted to create a composite tool (97, 98). The internal consistency was good (α = 0.88), with items demonstrating strong model fit (χ2 = 27.855; CFI = 0.978; TLI = 0.957; SRMR = 0.012) and average variance explained below 0.80 to demonstrate discriminant validity (102, 103). Intention to socialize was measured through a composite scale based on the literature (104). The internal consistency was good (α = 0.89), with items demonstrating strong model fit (χ2 = 92.562; CFI = 0.928; TLI = 0.906; SRMR = 0.051) and average variance explained below 0.80 to demonstrate discriminant validity. Digital literacy was measured through items adapted based on Ng (105). The internal consistency was good (α = 0.88), with items demonstrating strong model fit (χ2 = 48.957; CFI = 0.960; TLI = 0.921; SRMR = 0.038) and average variance explained below 0.80 to demonstrate discriminant validity. The confirmatory factor analysis (CFA) showed positive inter-dimensional relationships (between 0.47 and 0.69), and all items loaded to their latent construct between 0.70 and 0.84. The overall model showed good fit (χ2 = 383.149; CFI = 0.923; TLI = 0.923; SRMR = 0.059).

### Measurement model

A series of goodness of fit measures were examined (95, 106) including the chi-square/degree of freedom (χ2/df), Tucker-Lewis Index (TLI), Comparative Fit Index (CFI), Root Mean Residual (RMR), and the Root Mean Square Error of Approximation (RMSEA) (see Table 2). Hence, in this study, the measurement model was evaluated for single dimensionality, reliability, convergent validity, and discriminant validity.

**Table 2:** Goodness fit indices for the measurement model.

**Model**	**χ2/*df***	**CFI**	**TLI**	**RMR**	**RMSEA**
Target	≤ 5.0	≥0.90	≥0.90	≤ 0.09	≤ 0.05
Model 1 (Final model)	3.456	0.93	0.92	0.041	0.049
Model 2 (Independent constructs)	3.183	0.95	0.94	0.038	0.043
Model 3 (UTAUT removed)	3.147	0.94	0.91	0.022	0.036

### Validity and reliability

The study examined discriminant validity for social media usage implementation for teaching and learning in higher education over three criteria: first, the relationship index among constructs is <0.80 (95). Second, the AVE of every variable is equal to or <0.5. The AVE of every variable is better than the inter-construct correlations connected to that element (107). The measurements and the confirmatory factor examination outcomes factor loading of 0.5 or higher is satisfactory, Cronbach's Alpha (CA) 0.70, and the Composite Reliability (CR) 0.70 (95). In addition, the composite reliability values are shown, ranging from 0.84 to 0.90, all of which are better than the recommended value of 0.70. This includes the alpha values of CA between 0.72 and 0.90, apart from the total recommended value of 0.70. In addition, the AVE varies from 0.51 to 0.64; they are all equivalent or better than the suggested value of 0.50. Hence, it demonstrates that all factor loadings were important and exceeded 0.50, hence, achieving the recommended suggestions (95, 107). Table 3 provides a full overview.

**Table 3:** Overall of validity and reliability for students.

	**1**	**2**	**3**	**4**	**5**	**6**	**7**	**8**	**9**	**10**	**11**	**12**	**13**	**14**	**AVE**	**CR**	**CA**
Constructivist learning	0.57														0.59	0.88	0.78
Student intention	0.37	0.81													0.60	0.88	0.88
Digital connectivity	0.28	0.35	0.69												0.55	0.88	0.87
Perceived usefulness	0.34	0.29	0.28	0.77											0.56	0.86	0.86
Perceived ease of use	0.32	0.37	0.20	0.52	0.80										0.60	0.88	0.88
Behavioral intention	0.31	0.35	0.29	0.35	0.34	0.52									0.51	0.84	0.72
Performance expect	0.39	0.37	0.27	0.43	0.45	0.36	0.74								0.58	0.87	0.86
Effort expectancy	0.24	0.27	0.23	0.34	0.33	0.39	0.35	0.83							0.64	0.90	0.90
Actual social media	0.28	0.33	0.24	0.31	0.29	0.32	0.34	0.30	0.53						0.57	0.89	0.79
Intention to socialize	0.32	0.35	0.37	0.30	0.26	0.25	0.23	0.22	0.27	0.78					0.62	0.89	0.89
Digital literacy	0.30	0.32	0.29	0.25	0.22	0.26	0.24	0.28	0.26	0.34	0.75				0.60	0.88	0.88
Task-technology fit	0.35	0.35	0.39	0.31	0.26	0.32	0.28	0.21	0.29	0.47	0.33	0.72			0.60	0.88	0.88
Student satisfaction	0.40	0.34	0.29	0.27	0.22	0.30	0.23	0.25	0.23	0.35	0.31	0.34	0.66		0.53	0.87	0.87
Performance impact	0.35	0.33	0.33	0.31	0.29	0.32	0.31	0.28	0.31	0.33	0.32	0.44	0.34	0.63	0.51	0.84	0.81

### Hypothesis testing

**Figure 2:**
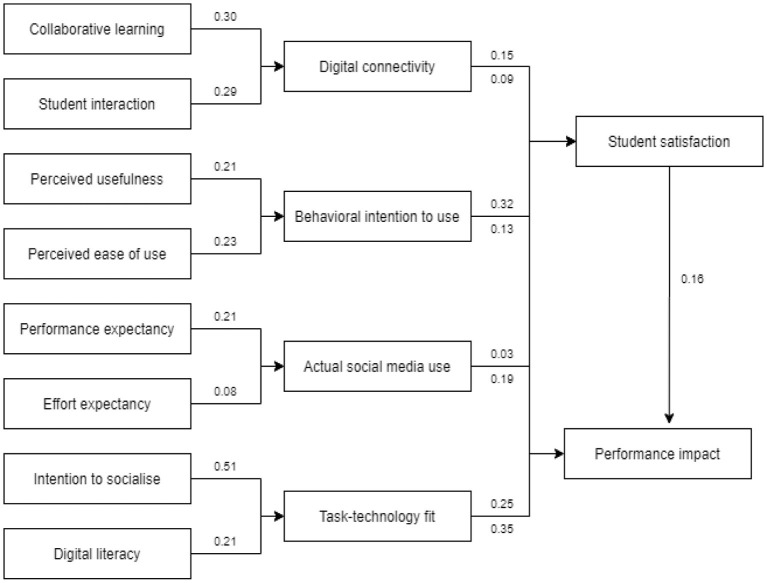
Results for the proposed model of all student groups.

As shown in Table 4, Figure 2, all hypotheses are accepted except for one, which is “no actual social media use between student groups for students' satisfaction”. The present sample shows that student groups do not have ASMU with peers leading to students' satisfaction for Digital connectivity (0.03-H12). Therefore, the hypothesis for every construct was greater than the other constructs. For example, the Hypothesis of intention to socialize on Task-technology fit (TTF) was shown to be positively and significantly related to Task-technology fit (TTF) to performance impact (PI) for adopting social media use during COVID-19 in higher education (β = 0.515, t = 13.087, *p* < 0.001) if being compared to its other hypothesis value (e.g., Task-technology fit (TTF) to Behavioral intention to use (β = 0.349, t = 8.547, *p* < 0.001). Another example is the Hypothesis Digital connectivity was shown to be positively and significantly related to performance impact (β= 0.265, t = 7.826, *p* < 0.001). While the lowest hypothesis value emerges on the path between effort expectancy (EEX) and Actual social media use (β= 0.082, t = 2.266, *p* < 0.001).

**Table 4:** Structural model for Hypothesis testing results.

**Hypothesis**	**Estimate**	**Standardized error**	**C.R**.	**Significance (*p*)**	**Result**
H1	0.30	0.05	5.54	0.000	Supported
H2	0.29	0.05	6.45	0.000	Supported
H3	0.21	0.04	5.01	0.000	Supported
H4	0.23	0.04	5.81	0.000	Supported
H5	0.21	0.04	5.61	0.000	Supported
H6	0.08	0.04	2.27	0.023	Supported
H7	0.52	0.04	13.09	0.000	Supported
H8	0.21	0.04	5.16	0.000	Supported
H9	0.156	0.04	4.41	0.000	Supported
H10	0.25	0.05	5.15	0.000	Supported
H11	0.345	0.04	8.55	0.000	Supported
H12	0.03	0.06	0.55	0.583	Unsupported
H13	0.20	0.05	4.18	0.000	Supported
H14	0.31	0.05	5.77	0.000	Supported
H15	0.267	0.03	7.83	0.000	Supported
H16	0.32	0.06	5.30	0.000	Supported
H17	0.13	0.05	2.61	0.009	Supported
H18	0.15	0.05	3.13	0.002	Supported
H19	0.09	0.04	2.38	0.017	Supported
H20	0.16	0.04	3.95	0.000	Supported

## Discussion

Our study results provide insight into student satisfaction and academic performance achievements and relationships with collaborative learning, interaction for learning, digital connectivity, perceived usefulness of social media, perceived ease of use of social media, behavioral intentions, performance expectancy, effort expectancy, actual social media use, intention to socialize, digital literacy, and task-technology fit. Moreover, the utilization of social media eases the context that is described throughout the behavioral intention of using it, digital connectivity, task-technology fit, and actual social media that could support users and students on social media. This eases the evolution to more doable models of teaching and learning requirements. Based on the conclusions and insights, the use of social networks can promote a favorable or supportive environment that is useful for learning, cooperation, and learning engagement. It can also increase performance expectancy and effort expectancy for actual social media use for learning in educational institutions. It develops the environment by fostering collaboration and contact amongst students as well as by facilitating discussion groups and the completion of work or research programs, which in turn boosts the influence of students on their performance (88, 108–110). In addition, social media is proved by advances in research abilities by trainers and the interchange of concepts among students for usage for behavioral and actual social media purposes to provide more utility than face-to-face (111).

This research contributes to literature through the suggestion of a model that integrates constructivist learning, unified theory of acceptance and usage of technology, and task-technology fit theories with the technology acceptance model, showing a useful model to recognize that five key implications. First, collaborative learning and student interaction for learning *via* social media impacts educational quality, increasing student satisfaction and academic performance. Second, perceived ease of use and perceived usefulness using social media effects on student behavioral intention to use social media for educational quality and actual social media usage, and it increases satisfaction and academic performance among students. Third, expected performance and effort from social media use influence actual social media usage among students as it improves their academic performance. Fourth, a student's intention to socialize online and their current digital literacy influence task-technology fit *via* social media use. It extends to improve their satisfaction and academic performance. Fifth, development of a theoretical model for social media usage in digital connectivity, task-technology fit, and other allied technologies. The study's contribution to the first model is combined with four theories, which are the unified theory of acceptance and usage of technology theory, constructivist learning theory, task-technology fit theory, and the technology acceptance model theory. The technology acceptance model also helps to use future social media for the purpose of providing greater learning and teaching outcomes.

The main practical consequences and contributions of the study are therefore obtained by addressing the research questions. First, the constructivist theory provided confirmation that it is an appropriate model for achieving collaboration for learning and interaction for learning to improve the quality of education among students, which in turn improves their educational performance in higher education. Second, the unified theory of acceptance and usage of technology provides evidence to model performance and effort expectancy and to increase actual social media usage among students, which consequentially increased student educational performance in higher education. Third, the technology acceptance model provided confirmation that it is an appropriate model to understand perceived usefulness and ease of use to improve behavioral intention to use among students, which in turn increases student educational performance in higher education. Fourth, the task-technology fit theory has been demonstrated to be appropriate to understand digital literacy and student intention to socialize to improve task-technology fit among students, which further increases their learning outcomes attainment in higher education. These are the significant theoretical contributions to previous studies on these theoretical areas, which previously did not identify the impacts of using social media on educational quality, actual social media usage, and task-technology fit (81, 112, 113).

## Conclusion and future work

The findings of this study improve the understanding of the actual collaborative learning and student interaction with digital connectivity, a particularly important considering during the pandemic context (2). The results also showed that perceived usefulness and ease of use increase the behavioral intention to use social media through the quality of education and actual social media usage for education; these factors finally affect their satisfaction and academic performance. Similarly, the findings also showed that performance and effort expectancy, and in turn, actual social media use for education, eventually affect a person's academic performance and also their social and technological characteristics to increase task-technology fit. Furthermore, the outcomes of the study showed that student behavioral intention to use social media positively affects their educational quality and ASMU for learning in educational institutions, and ultimately, their students' satisfaction and academic performance. The use of the technology acceptance model, unified theory of acceptance and usage of technology, constructivism theory, and task-technology fit in examining students' educational quality for behavioral intention to utilize social media and actual social media use, and also task-technology fit to actual social media use to improve the students' satisfaction and academic performance impact in higher education is also confirmed. Both theories are used for the measurement of students' satisfaction and academic performance in higher education, which has yet to be touched by several studies in higher education contexts. Overall, the educational quality and task-technology fit *via* social media boost students' motivation for teaching and learning and enables conversation with peers. The study provides new consequences, but also has some drawbacks. One of the limitations is that the study sample size is limited by data collection. As a result, the study's findings do not necessarily imply behavioral intention in other educational levels (e.g., high school). Future work is suggested to increase data collection from universities or school students in other states or repeat the research in other provinces rather than Malaysia to strengthen the understanding of how these theories relate in different geographic jurisdictions.

## Data availability statement

The original contributions presented in the study are included in the article/supplementary material, further inquiries can be directed to the corresponding author.

## Author contributions

AA-R, AS, and EW: conceptualization, methodology, resources, data curation, and project administration. AA-R, WA-R, and IA: software. AA-R, AS, EW, JC, and IA: validation. AA-R, AS, EW, and WA-R: formal analysis. AA-R: investigation. AA-R, WA-R, and AS: writing-original draft preparation and writing-review and editing. AA-R, AS, EW, WA-R, IA, and JC: visualization. AS, WA-R, and EW: supervision. All authors have read and agreed to the published version of the manuscript.

## Funding

This work was supported by the Deanship of Scientific Research, Vice Presidency for Graduate Studies and Scientific Research, King Faisal University, Saudi Arabia [Grant No. 1558]. Also, Communication of this research is made possible through monetary assistance by Universiti Tun Hussein Onn Malaysia and the UTHM Publisher's Office via Publication Fund E15216.

## Conflict of interest

The authors declare that the research was conducted in the absence of any commercial or financial relationships that could be construed as a potential conflict of interest.

## Publisher's note

All claims expressed in this article are solely those of the authors and do not necessarily represent those of their affiliated organizations, or those of the publisher, the editors and the reviewers. Any product that may be evaluated in this article, or claim that may be made by its manufacturer, is not guaranteed or endorsed by the publisher.
